# CaS:Mn_1-x_Pb_x_ Luminescent Material Production from Phosphogypsum

**DOI:** 10.3390/molecules31010040

**Published:** 2025-12-22

**Authors:** Zlatislava D. Khliyan, Nina P. Shabelskaya, Oleg A. Medennikov, Marina A. Egorova, Darya V. Yakhonova, Galina N. Zemchenko, Yuliya A. Gaidukova, Vera A. Baranova, Asatullo M. Radjabov, Angelika V. Serik

**Affiliations:** Department of Ecology and Industrial Safety, Faculty of Technology, Platov South-Russian State Polytechnic University (NPI), Novocherkassk 346428, Russia; zlata.tkachenko.98@mail.ru (Z.D.K.); nina_shabelskaya@mail.ru (N.P.S.); monomors@yandex.ru (O.A.M.); dasha29yahonova08@gmail.com (D.V.Y.); gnzem77@yandex.ru (G.N.Z.); u.vera20@yandex.ru (V.A.B.); rajabov.asadullo@mail.ru (A.M.R.); anzelika2027@gmail.com (A.V.S.)

**Keywords:** phosphogypsum processing, calcium sulfide solution, manganese doping, lead doping, ultraviolet pigments

## Abstract

This paper explores the feasibility of producing manganese- and lead-doped luminescent materials from phosphogypsum. For the first time, orange- and red-emitting ultraviolet pigments were obtained using a sulfide matrix reduced from phosphogypsum. The resulting materials were characterized using X-ray diffraction (XRD), transmission electron microscopy, Fourier transform infrared spectroscopy, elemental analysis, and X-ray photoelectron spectroscopy (XPS). Doping with manganese or lead cations is shown to produce luminophores whose luminescence shifts from orange to red–orange under UV radiation as lead cations replace manganese cations in the CaS:Mn_1-x_Pb_x_ solid solution. A sharp increase in red luminescence intensity was observed for CaS: Mn luminophores when they were irradiated with short-wavelength ultraviolet radiation. These results open up broad possibilities for using phosphogypsum, a high production volume (HPV) chemical waste product, to produce highly innovative products.

## 1. Introduction

In modern society, there is a high demand for various products that glow when exposed to ultraviolet radiation. Ultraviolet (UV) pigments, as well as paints and varnishes based on them, are in steady demand for use as counterfeit markers [1] and daylight sources [2]. UV pigments can be induced by long-wavelength (UVA, 400–320 nm), medium-wavelength (UVB, 320–280 nm), and short-wavelength (UVC, 280–180 nm) radiation. Long-term research [3,4,5] demonstrates the pronounced bactericidal effect of short-wavelength ultraviolet radiation. This property is the basis for room disinfection irradiator development, for example, those used during the spread of COVID-19. However, short-wavelength radiation leads to skin aging [1,3] and is harmful to human health. In this regard, the search for inexpensive sensors capable of signaling the presence of such harmful radiation is urgent.

Sulfides are a common matrix for producing luminescent materials [2,6,7,8,9,10,11,12,13]. Sulfides of lanthanides [7,8], *d*-elements [2,6,7,8,13], and alkaline earth elements [10,14] successfully serve as host matrices. Lanthanides [2,8,9,10] and *d*-elements [2,13] are often used as doping cations. Changing the type of doping cation leads to a change in the luminescence color. For example, luminophores of the following colors and compositions have been obtained: yellow and orange ZnS:Mn^2+^ [15,16,17]; blue and green ZnS:Cu^2+^ [15]; orange and blue ZnS:Ni^2+^ [2]; blue–green ZnS:Cu, Al, ZnS:Cu,Cl [17,18]; orange and red ZnS:Eu^3+^ [19,20]; dark red ZnS:Eu^2+^ [19]; green ZnS:Tb^3+^ [2]; red ZnS:Sm [19]; blue ZnS:Ce^3+^ [15,21]. Replacement of the cation in the host matrix is also accompanied by a change in the color of the luminescence. For example, CaS: Ce is green, while SrS: Ce is blue [14]. Calcium sulfide is often used as a phosphor matrix [11,14].

Luminescent material synthesis is most often carried out using the solid-phase reaction method at fairly high temperatures (1000–1250 °C) [8,15,19,21]; chemical co-precipitation methods [2,15,21] and the sol-gel method [19,20] are also used.

Phosphogypsum is a waste product from the phosphoric acid production of apatite raw materials [22]. Its huge waste heaps can lead to environmental pollution. Therefore, numerous attempts are being made worldwide to recycle phosphogypsum. It is used, for example, in the production of binders [23,24], road construction [25], heat storage materials [26], and fertilizer production [27].

In this study, we report on the feasibility of phosphogypsum recycling to produce luminescent materials with various emission colors (orange, red), based on a calcium sulfide matrix. To the best of our knowledge, such a study has not yet been conducted. The synthesis of luminescent materials is usually carried out from very high-purity starting materials, which results in high costs of the resulting products. We offer products with high added value, synthesized by a simple method from readily available raw materials.

## 2. Results and Discussion

**Figure 1:**
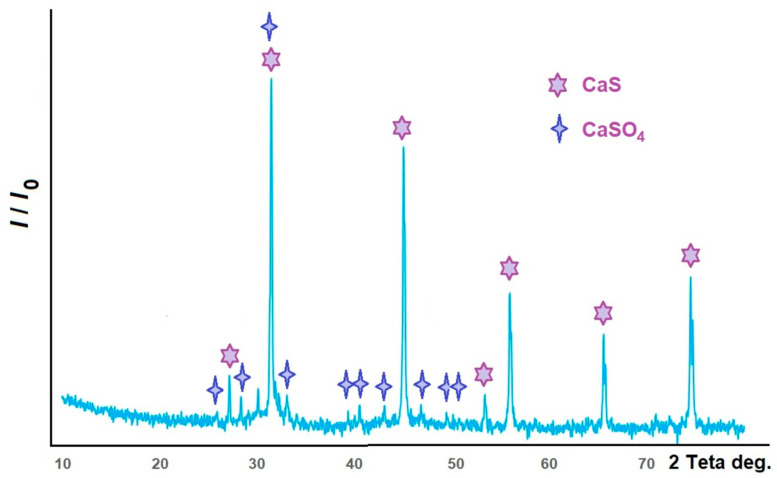
X-ray diffraction analysis of a CaS: Mn sample.

The X-ray diffraction pattern of one of the samples (doped with manganese) is shown in Figure 1. Analysis of the obtained data indicates that calcium sulfate was almost completely converted to sulfide (the CaS/CaSO_4_ phase ratio can be estimated at 92–93:7–8).

Only peaks belonging to calcium sulfide and calcium sulfate were identified in the samples using X-ray phase analysis. The main lines characterizing calcium sulfide have maxima at the following values of double angles (indices of interplanar distances): 27.7 (111); 31.4 (200); 45.1 (220); 53.4 (311); 56.0 (222); 65.6 (400); and 74.6 (420). The calcium sulfide phase (PDF Number: 010-75-0261) is crystallized in the cubic syngony; the calculated lattice parameter of *a* = 0.5694 nm is in good agreement with the tabulated value of *a* = 0.5684 nm. Thin clear peaks indicate that the phases are well crystallized. The sample also contains a phase of anhydrous calcium sulfate (PDF Number: 010-86-2270, orthorhombic syngony). The main lines characterizing calcium sulfate have maxima at the following double angles (interplanar spacing indices): 25.5 (020); 28.6 (002); 31.4 (012); 32.0 (121); 38.6 (202); 40.8 (212); 43.3 (131); 48.7 (032); and 49.1 (321). Some of the lines of these phases coincide. Manganese and/or lead compounds are not identified in the X-ray diffraction patterns due to their low contents.

**Figure 2:**
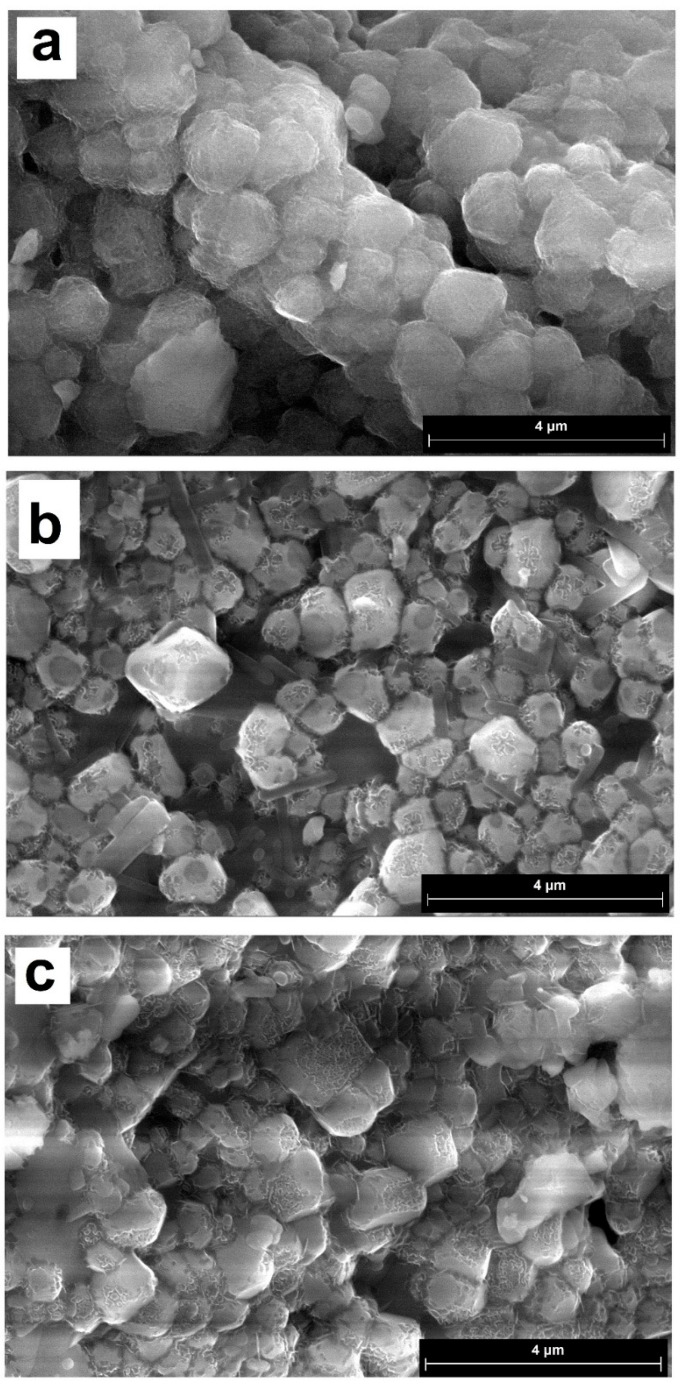
SEM images of the synthesized materials: (**a**)—CaS: Mn; (**b**)—CaS: Mn/Pb; and (**c**)—CaS: Pb.

Figure 2 shows micrographs of the synthesized materials. Unlike calcium sulfate, which forms plate-shaped crystals, reduced phosphogypsum is represented by cubic crystals. In compositions containing the lead cation, characteristic four-petal flower-like patterns are visible on the surface. Crystal sizes for CaS:Mn are 0.26–1.62 microns, for CaS:Pb are 0.57–1.14 microns, and for CaS:Mn/Pb are 0.26–1.21 microns. When switching from CaS:Mn to CaS:Pb, the formation of smaller crystals was observed. It should also be noted that, in CaS:Mn/Pb, individual crystals are more pronounced; for manganese-only or lead-only doped samples, they are more likely to form clusters.

**Figure 3:**
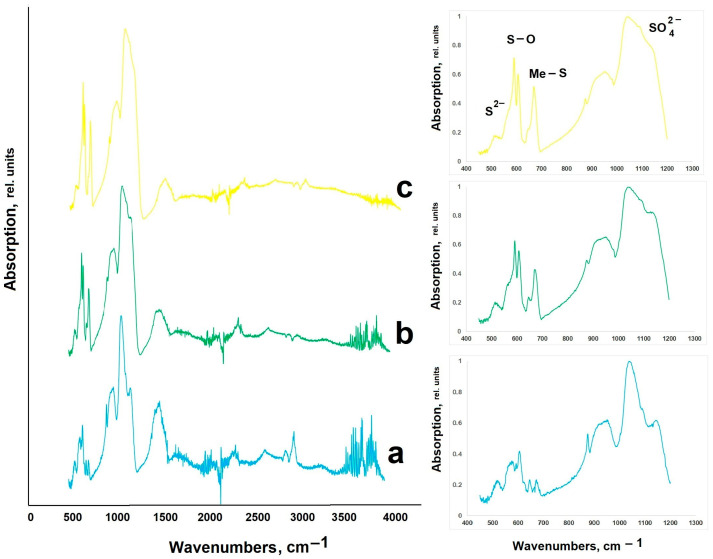
IR spectra of CaS:Mn_1-x_Pb_x_: (**a**)—CaS:Mn; (**b**)—CaS:Mn/Pb; and (**c**)—CaS:Pb.

**Figure 4:**
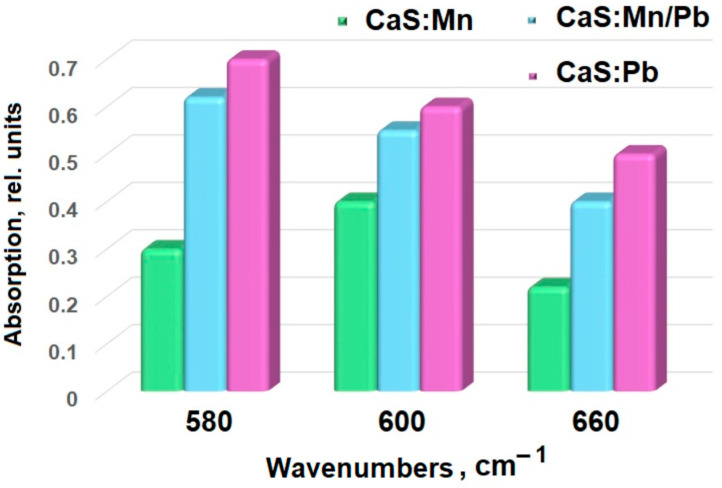
Several IR spectral lines’ intensity dependences.

The samples’ compositional data were confirmed by infrared spectroscopy (Figure 3 and Figure 4).

Valence vibrations of sulfides (Figure 3) are located in the low-wavelength range [28], so the 650–680 cm^−1^ band characterizes Me-S vibrations [29,30]. As manganese ions are replaced by lead ions, an increase in the line intensity is observed (from 0.22 for CaS:Mn and 0.40 for CaS:Mn/Pb to 0.50 for CaS:Pb, Table 1, Figure 4), while a shift of the peak towards shorter wavelengths is noted (from 678 cm^−1^ for CaS:Mn and 675 cm^−1^ for CaS:Mn/Pb to 672 cm^−1^ for CaS:Pb). This effect may be associated with an increase in the polarity of the Me-S bond (Me = Mn, Pb) upon successive substitution of Mn by Pb. The lines 550–600 and 1000–1100 cm^−1^ can be attributed to vibrations of the S-O group [31,32,33] in sulfates. Lines in the region of 1450 and 3500 cm^−1^ are attributed [31] to vibrations of O-H groups.

**Figure 5:**
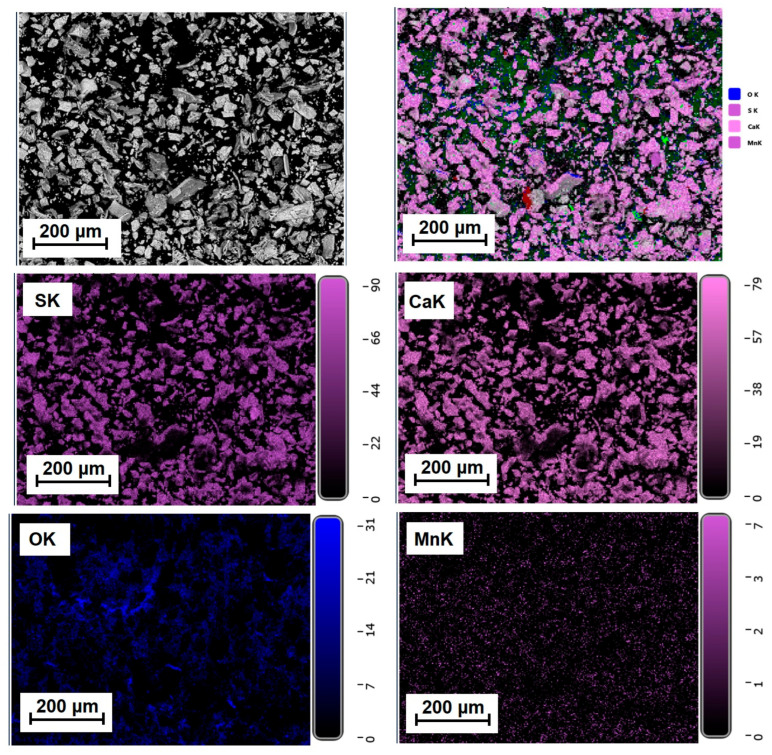
Distribution of major elements in a CaS:Mn sample.

**Figure 6:**
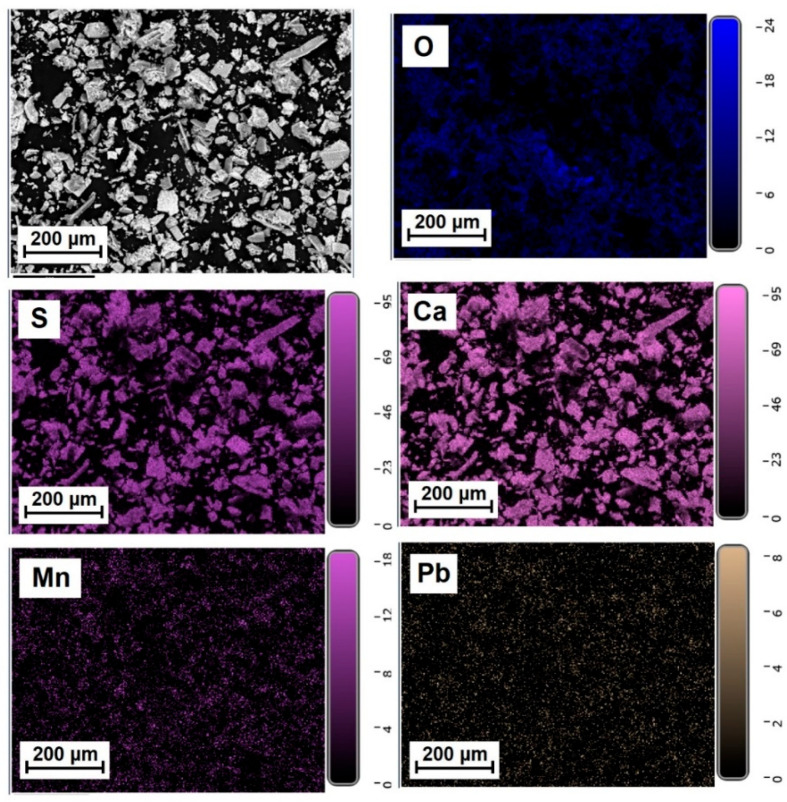
Distribution of major elements in a CaS:Mn/Pb sample.

**Figure 7:**
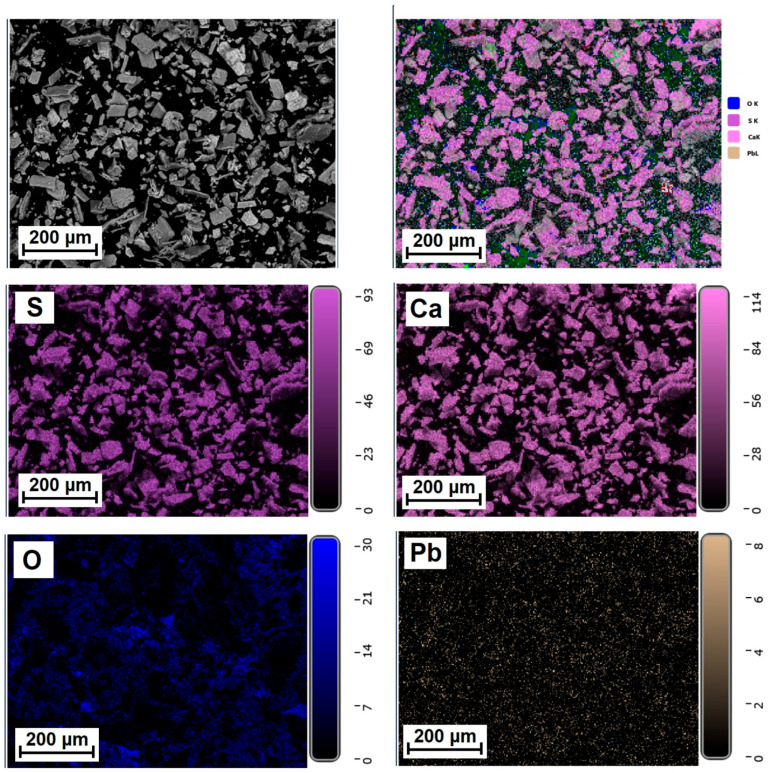
Distribution of major elements in CaS:Pb sample.

Figure 5, Figure 6 and Figure 7 show the elemental distribution in a reduced phosphogypsum sample based on elemental analysis. The analysis data indicate a uniform distribution of elements throughout the sample, with the exception of oxygen. Areas with higher oxygen content are visible. These data indicate the formation of a composite material whose main phase contains calcium sulfate and calcium sulfide.

**Figure 8:**
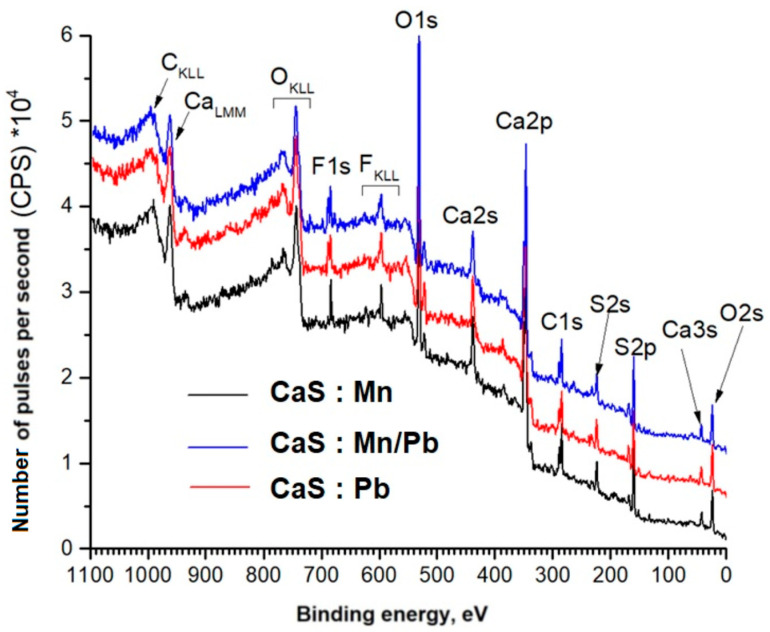
Overview spectra of phosphogypsum samples.

Panoramic XPS spectra of phosphogypsum samples, indicating the lines of detected chemical elements, are shown in Figure 8. We assume that a small amount of fluorine (its lines are present in Figure 8) was added to the sample during sample preparation. Notably, the concentration of doping cations in the surface layers is higher when they are present together in the sample (Table 2).

**Figure 9:**
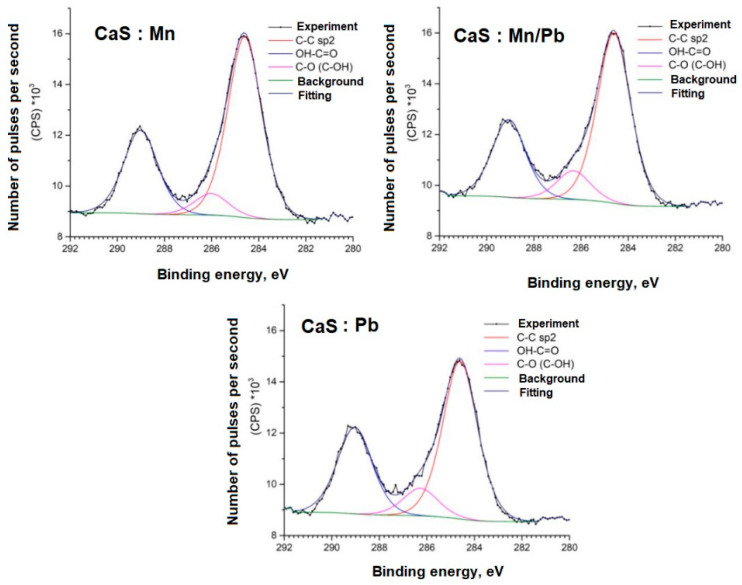
C1s spectra of samples.

**Figure 10:**
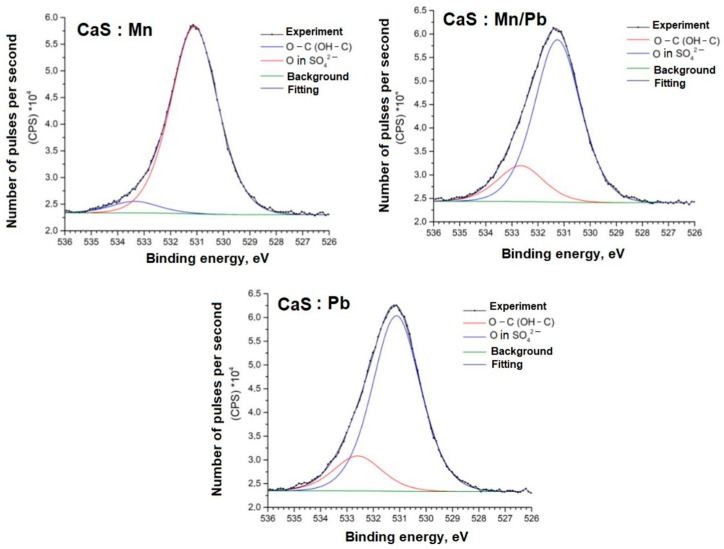
O1s spectra of samples.

**Figure 11:**
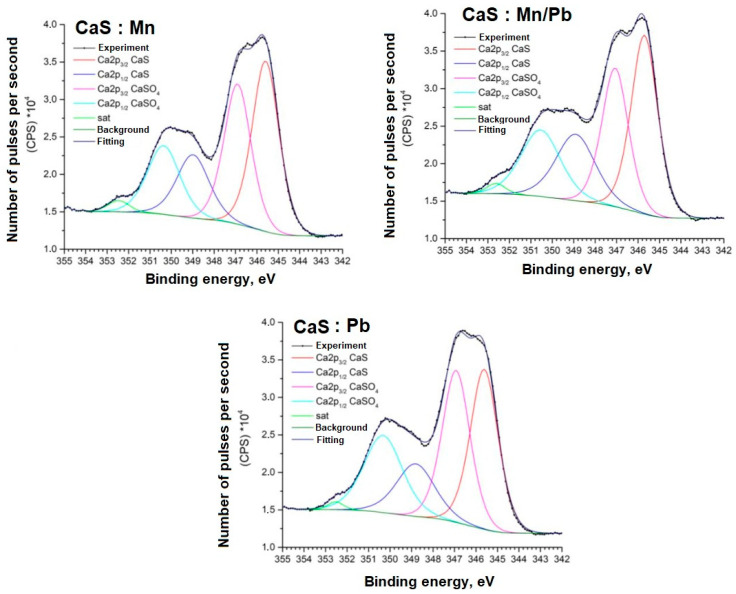
Ca2p spectra of samples.

**Figure 12:**
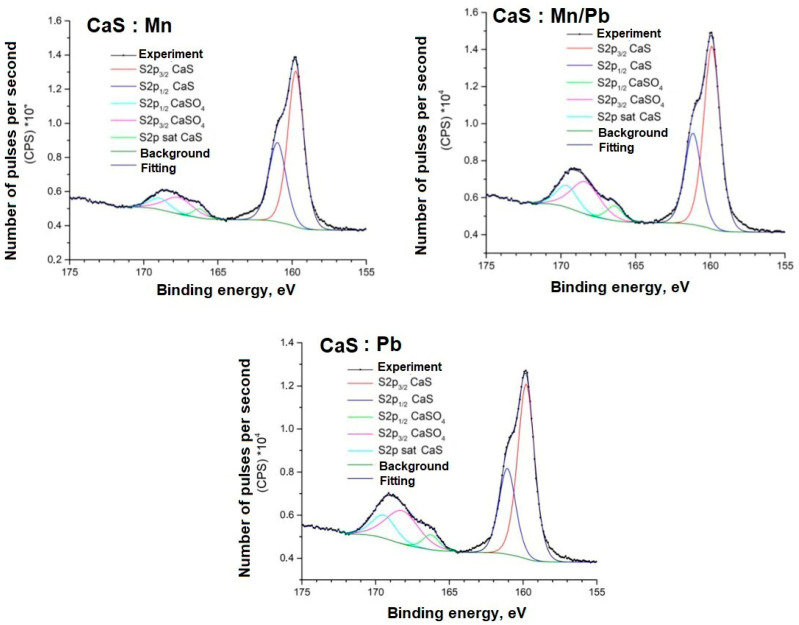
S2p spectra of samples.

**Figure 13:**
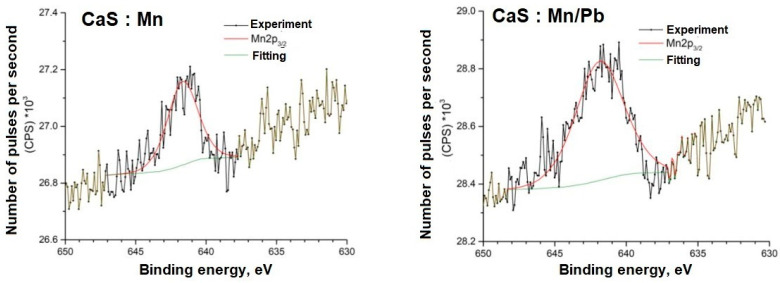
Mn2p spectra of samples.

**Figure 14:**
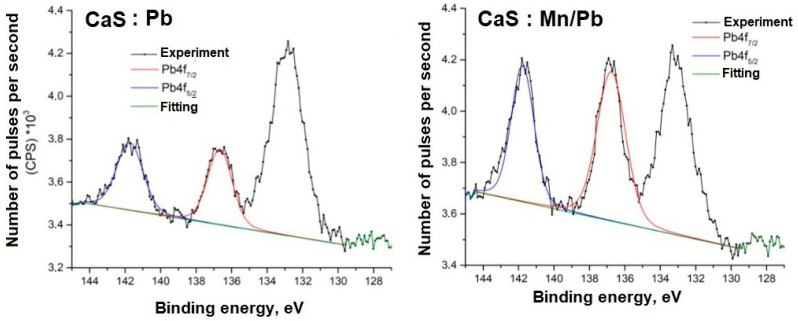
Pb4f spectra of samples.

Detailed spectra of the samples and the parameters for their decompositions into components are presented below; the corresponding chemical bonds are indicated (Figure 9, Figure 10, Figure 11, Figure 12, Figure 13 and Figure 14).

The C1s spectra of carbon show an increased content of OH-C=O bonds for adsorbed carbon, which may be due to the specifics of the sample synthesis.

The O1s spectra, in addition to the oxygen bonds in the SO_4_^2−^ groups, show lines (Eb = 532–533 eV) associated with carbon. This may be due to the synthesis specifics and incomplete removal of the reducing agent from the system.

The positions and intensity ratios of the Ca2p and S2p lines of the samples correspond to those of calcium sulfide (CaS) and sulfate (CaSO_4_), indicating that the powders are a mixture of two phases based on Ca and S, as confirmed by X-ray diffraction analysis and FTIR spectroscopy. The ratio of sulfide to sulfate is 93/7.

The energy positions of the Mn2p and Pb4f spectra indicate their associations in the sulfide.

Previously, we [34] established the possibility of producing a luminescent material through the thermal reduction of phosphogypsum. The resulting material emitted a yellow glow (with a wavelength of approximately 585 nm) when irradiated with ultraviolet radiation at a wavelength of 390 nm.

**Figure 15:**
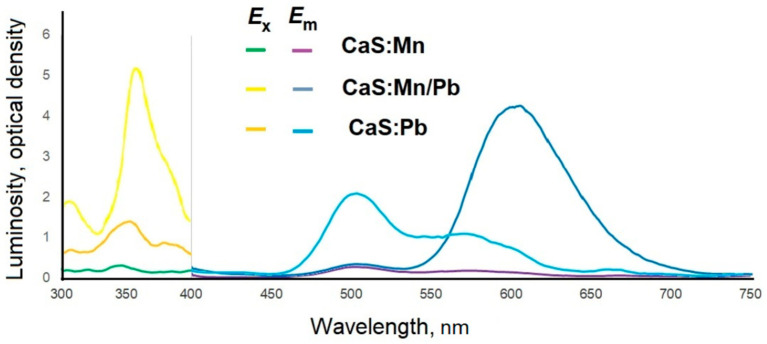
Excitation (*E*_x_) and emission (*E*_m_) spectra of CaS:Mn_1-x_Pb_x_ samples.

Doping with manganese cations shifted the excitation wavelength to 350 nm; the emission was recorded in the range of 450–680 nm, with peaks at 505, 550, and 670 nm, with a luminescence intensity of 0.3 relative units for CaS:Mn and 2 relative units for CaS:Pb. Joint doping with manganese and lead cations resulted in a significant (3–20 times compared to CaS:Mn (intensity 0.15 relative units) and CaS:Pb (intensity 1.0 relative units) CaS:Mn/Pb (intensity 3.1 relative units)) increase in the luminescence intensity and a shift of the luminescence peak to the red region (625 nm) (Figure 15).

**Figure 16:**
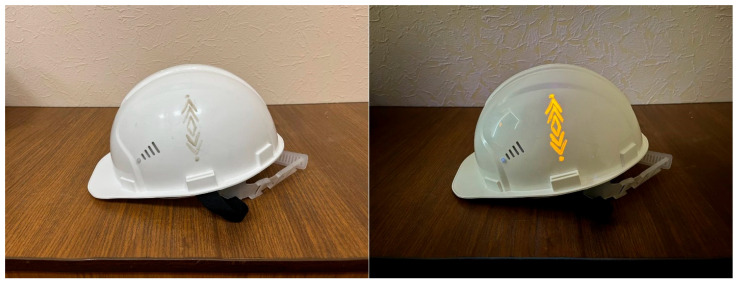
Photograph of a product decorated with the developed pigment under natural lighting (**left**) and under ultraviolet illumination (**right**).

Paints and varnishes were obtained using the synthesized pigments. Nitrocellulose varnish and colorless polyvinyl chloride varnish were used as the bases. We injected 20% (wt.) synthesized pigment CaS:Mn/Pb and 25% (by weight) chalk. The luminescence intensity of the phosphor in the powder was 3.1 relative units, and in the coating was 3.01 relative units, with almost no losses. Figure 16 shows a photograph of a product decorated with the developed materials under normal lighting and long-wavelength ultraviolet light.

**Figure 17:**
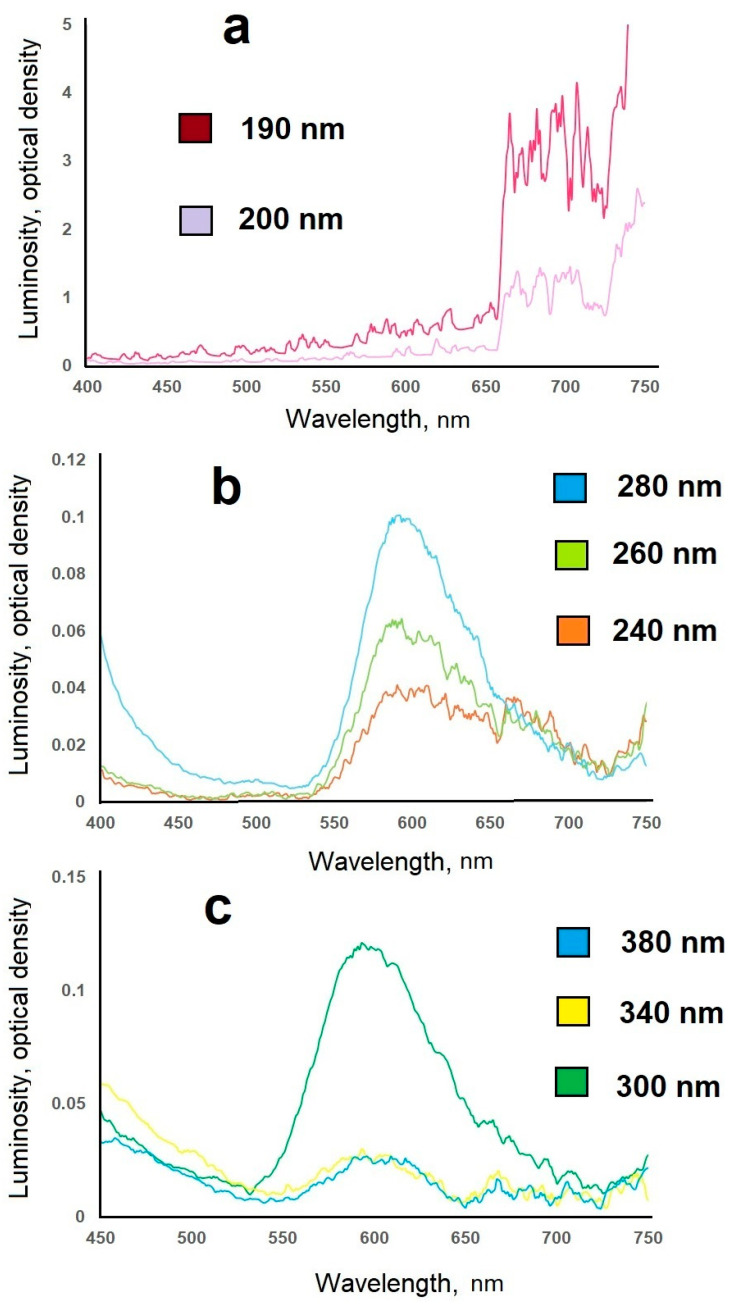
Luminescence spectra of a CaS: Mn sample with different excitation wavelengths: (**a**)—far-ultraviolet, (**b**)—mid-ultraviolet, (**c**)—near-ultraviolet.

For manganese-doped samples, a sharp increase in luminescence intensity was observed upon irradiation with far-ultraviolet radiation (Figure 17).

The synthesized CaS:Mn samples were irradiated with near-ultraviolet radiation (300–400 nm), although the emission was weak (0.024–0.064 relative units), peaking in the orange region at 600 nm. When the synthesized samples were irradiated with mid-ultraviolet radiation (200–300 nm), the emission was weak (0.02–0.1 relative units), peaking in the red–orange region at 620 nm. When the synthesized samples were irradiated with far-ultraviolet radiation (122–200 nm), the emission was several times amplified (3.71 relative units, up to 40-fold), shifting toward the red region of the spectrum (665–710 nm).

**Figure 18:**
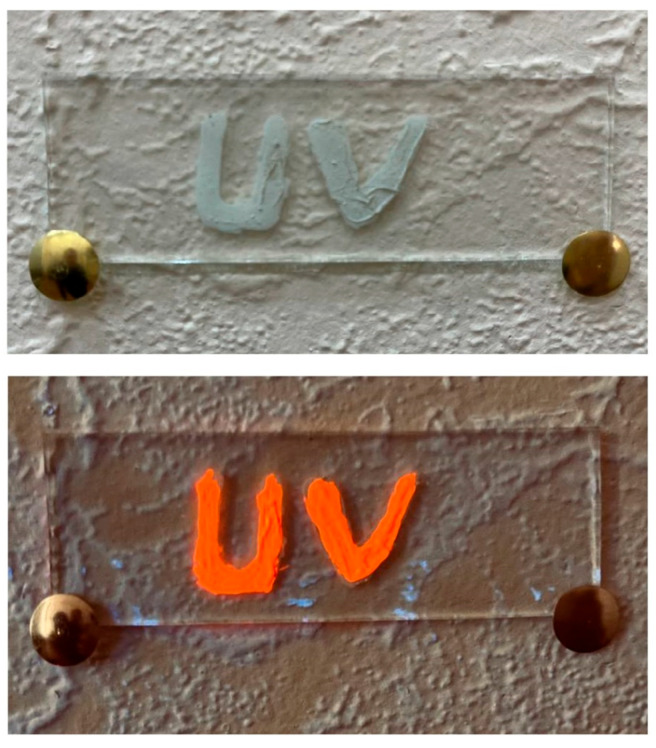
Example of using the developed materials as a sensor (upper picture—normal lighting, lower picture—ultraviolet lighting).

This experimentally established fact is of fundamental importance: the resulting materials can be used as affordable sensors for detecting short-wave ultraviolet radiation (Figure 18), which has an extremely harmful effect on the human body.

It is especially worth emphasizing that the luminophores were obtained by recycling chemical production waste, without the use of high-purity reagents. Our research opens up broad opportunities for recycling industrial waste to produce sought-after high-tech products.

## 3. Materials and Methods

### 3.1. Materials

Agricultural phosphogypsum, containing 99% (wt) calcium sulfate dihydrate CaSO_4_∙2H_2_O, was used to obtain calcium sulfide. To introduce the doping cations, a solution of manganese or lead salts with a cation content of 5 g/L was prepared. Chemically pure MnSO_4_·7H_2_O and Pb(NO_3_)_2_, along with deionized water, were used to prepare the salt solutions. Potato starch, with a main component content of 99.5% (wt), was used as the reducing agent.

### 3.2. Luminophore Synthesis

A solution of manganese(II) (6 mL) (or lead(II) (30 mL)) salt was added to 17.2 g of phosphogypsum, and the mixture was dried to constant weight at 100 °C (for approximately 2 h). When a sample containing both doping cations was prepared, the manganese cation (4 mL) was added first and dried, then the lead cation (30 mL) was added, and the sample was dried again; 7 g of starch was added to the samples and thoroughly ground in a mortar until a homogeneous mixture was formed.

**Figure 19:**
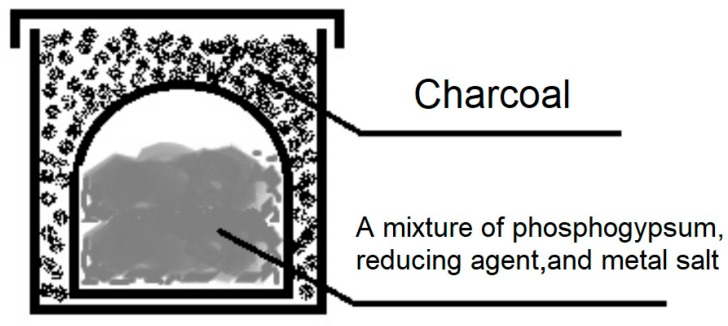
Schematic diagram of the loading method.

The samples were placed in alundum crucibles using a crucible-in-crucible system, covered with carbon, and covered with lids (Figure 19).

After this, they were placed in the working space of a muffle furnace, where they were heat-treated. Heating was carried out at a rate of 13 °C/min and held at the maximum heat-treating temperature of 1000 °C for 60 min. The proposed method can be considered a traditional production method.

### 3.3. Characterization

To characterize the resulting composite materials, various methods were used, i.e., X-ray diffraction (XRD), transmission electron microscopy with elemental analysis, Fourier transform infrared spectroscopy, and X-ray photoelectron spectroscopy (XPS).

The phase composition was studied on an ARL X’TRA X-ray diffractometer (Thermo Fisher Scientific (Ecublens) SARL, Ecublens, Switzerland) (using monochromatic Cu-Kα radiation), using point-by-point scanning (0.01° step, 2 s accumulation time per point) over a 2θ range from 5° to 90°. The result of using the RIR method, using the PDXL2 program and the PDF2 database for analyzing X-ray diffraction data, is provided in the additional file AF.

Elemental analysis was performed using a Quattro S SEM (Thermo Fisher Scientific, Waltham, MA, USA) equipped with an Octane Elite Plus (EDAX) EDS microanalyzer.

The IR spectra of the samples were obtained using a Fourier Spectrum Two (Perkin-Elmer, Waltham, MA, USA) instrument, the frequency range of which varied from 400 to 4000 cm^−1^. The final processing of the spectra was carried out using Spectrum software, Micro-Cap 12.

X-ray photoelectron spectroscopy spectra were performed on a SPECS device (“SPECS GmbH—Surface Analysis and Computer Technology”, Berlin, Germany). For more information about the sample preparation process and survey parameters, see [34].

### 3.4. Study of Luminescent Properties

Excitation and luminescence spectra were recorded using an SM 2203Z Spectrofluorometer (JSC Spectroscopy, Optics and Lasers—Avant-garde Developments, Minsk, Belarus) in the excitation wavelength range of 190–380 nm and emission wavelength range of 400–750 nm.

## 4. Conclusions

For the first time, the possibility of producing orange and red UV pigments from phosphogypsum has been demonstrated. Calcium sulfide matrices have been shown to form during thermal treatment of phosphogypsum in the presence of a reducing agent. When doped with manganese or lead cations, luminophores are formed whose luminescence shifts from orange to red–orange regions under UV irradiation as lead cations replace manganese cations in the CaS:Mn_1-x_Pb_x_ solid solution.

For CaS: Mn luminophores, a sharp increase in luminescence intensity in the red region was observed when the sample was irradiated with short-wavelength UV radiation.

These results open up broad possibilities for using phosphogypsum, a high production volume (HPV) chemical waste product, to produce highly innovative products.

## Figures and Tables

**Table 1:** FTIR spectra of CaS:Mn1-xPbx analysis results.

Sample	Intensity, Rel. Units, at Wave Numbers (cm^−1^)						
	520	580	600	660	880	950	1040
CaS: Mn	0.22	0.30	0.40	0.22	0.55	0.6	1.0
CaS: Mn/Pb	0.20	0.62	0.55	0.40	0.50	0.65	1.0
CaS: Pb	0.19	0.70	0.60	0.50	0.41	0.60	1.0

**Table 2:** Element concentrations in the surface layers of samples, according to XPS data (at%).

Sample	C	O	Ca	S	Mn	Pb
CaS:Mn	24.3	35.4	23.6	16.6	0.1	-
CaS:Mn/Pb	21.3	36.6	23.3	18.2	0.22	0.48
CaS:Pb	21.2	39.4	23.0	16.1	-	0.28

## Data Availability

The original contributions presented in this study are included in the article.
